# Effects of ATP9A on Extracellular Vesicle Release and Exosomal Lipid Composition

**DOI:** 10.1155/2020/8865499

**Published:** 2020-10-29

**Authors:** Xiao Xu, Limei Xu, Peng Zhang, Kan Ouyang, Yin Xiao, Jianyi Xiong, Daping Wang, Yujie Liang, Li Duan

**Affiliations:** ^1^Department of Orthopedics, Shenzhen Intelligent Orthopaedics and Biomedical Innovation Platform, Guangdong Artificial Intelligence Biomedical Innovation Platform, Shenzhen Second People's Hospital, The First Affiliated Hospital of Shenzhen University Health Science Center, Shenzhen, Guangdong 518035, China; ^2^Shenzhen Institute of Advanced Technology, Chinese Academy of Sciences, Shenzhen, Guangdong 518055, China; ^3^Institute of Health and Biomedical Innovation, Faculty of Science and Engineering, Queensland University of Technology, Kelvin Grove Campus, Brisbane, QLD 4059, Australia; ^4^Shenzhen Kangning Hospital, Shenzhen Mental Health Center, Shenzhen, Guangdong 518020, China

## Abstract

Numerous biological processes are regulated by the intercellular communications arising from extracellular vesicles (EVs) released from cells. However, the mechanisms that regulate the quantity of EV discharged have yet to be understood. While it is known that ATP9A, a P4-ATPase, is involved in endosomal recycling, it is not clear whether it also contributes to the release of EVs and the makeup of exosomal lipids. This study is aimed at exploring the role of human ATP9A in the process of EV release and, further, to analyze the profiles of EV lipids regulated by ATP9A. Our results demonstrate that ATP9A is located in both the intracellular compartments and the plasma membrane. The percentage of ceramides and sphingosine was found to be significantly greater in the control cells than in the ATP9A overexpression and ATP9A knockout groups. However, EV release was greater in ATP9A knockout cells, indicating that ATP9A inhibits the release of EVs. This study revealed the effects of ATP9A on the release of EVs and the lipid composition of exosomes.

## 1. Introduction

As extracellular vesicles (EVs) released from cells are mediators of intercellular communications, they can transmit information between cells and are involved in tissue development and disease progression [1, 2]. EVs are categorized according to diameter, which ranges from 50 to 1000 nm. Based on the formation of EVs, they are categorized as either ectosomes or exosomes [3]. Ectosomes are created directly by the plasma membrane forming outward buds. Exosomes, however, are combination products of multivesicular endosomes (MVEs) fusing with the plasma membrane which are then discharged into extracellular space [4, 5].

One distinctive characteristic of eukaryotic cells is the composition of phospholipids in the plasma membrane, which are not distributed symmetrically. This has long been recognized as a potential influence on the release of vesicles. The distribution of phospholipid species differs in the two leaflets of biological membranes. The leaflet exposed to cytosol consists of phosphatidylserine (PS) and phosphatidylethanolamine (PE), whereas the exoplasmic leaflet contains mostly phosphatidylcholine (PC) and sphingomyelin [6]. This asymmetric phospholipid distribution is an important aspect in numerous diseases, such as cancer, cardiovascular disease, hepatic steatosis, and metabolic disorders. The asymmetric distribution is also involved in many physiological processes, including apoptosis, blood coagulation, apoptosis, cell signaling, cell and organelle morphology, host-virus interactions, intracellular vesicle trafficking, membrane permeability and stability, and regulation of membrane proteins [7, 8].

Usually, ATP-dependent lipid transporters are responsible for maintaining homeostasis of the phospholipid distribution; however, PS can be redistributed to the exoplasmic leaflet in response to pathophysiological conditions. This has been observed in platelet activation, in exosome release from tumor tissues, and in the majority of immune cell types, such as activated B cells, bone marrow-derived monocytes, dendritic cells, and macrophages [9].

The asymmetry of lipids is maintained actively by a variety of transporter families [10, 11]. One particular family is the P-type ATPase family, and the members of its largest subfamily, P4-ATPase, are known as flippases [7, 12]. These ATP-dependent enzymes relocate certain phospholipids away from the membrane's outer leaflet and toward the inner leaflet. This maintains the membrane's lipid asymmetry, which has been highlighted previously to be critical to several cellular processes. Mounting evidence indicates that in eukaryotic cells, P4-ATPases play a key role in the biogenesis of the transport vesicles involved in the biosynthetic and endocytic pathways [11].

Models characterizing the role of P4-ATPases in lipid translocation pathways have been informed by site-directed mutagenesis and structural modeling studies [13, 14]; however, the fundamental features of the transport pathway, the electrogenic properties of the flippases, and the mechanisms by which flipping occurs remain unclear. Using TAT-5, the P4-ATPase of *Caenorhabditis elegans*, Wehman et al. showed that the enzyme was involved in the release of EVs and maintenance of PE asymmetry [15]. Whereas, human ATP9A and ATP9B, which are orthologous to TAT-5, are primarily found inside intracellular compartments [16]. ATP9A is recognized as a regulator for endosomal recycling [17]; whether it contributes to EV release and the lipid profiles of exosomes has not yet been determined. It is well-known that during cell division, fusion, and death, cells regulate PE asymmetry and EV release [18]. Therefore, we hypothesized that ATP9 is involved in plasma membrane biosynthesis, cell outward budding, and EV release. This study examines the effects of ATP9A on the composition of exosomal lipids and the release of EVs in a human embryonic kidney 293 (HEK293) cell line.

## 2. Materials and Methods

### 2.1. Cell Culture

DMEM medium (DMEM; Life Technologies) plus 10% fetal bovine serum (FBS, Gibco) supplement was used to sustain the HEK293 cell line at 37°C and 5% CO_2_. Synovial fluid-derived human mesenchymal stem cells (SF-MSCs) were cultured in MesenGro Chemically Defined Medium.

### 2.2. Overexpression of ATP9A

Control for this study was provided by the lentivirus empty vector, pLenti-C-RFP, which does not contain any human genes. The 293T cells were variously transfected with pLenti-C-RFP, pMD2.G, and psPAX2. After 48 h, lentivirus was harvested and used to infect MSCs, followed by puromycin selection (used as plv-NC group). The whole length of the ATP9A gene was subcloned into the destination pLenti-C-RFP plasmids. pLenti ATP9A-RFP plasmids were then cotransfected into HEK293T cells along with psPAX2 and pMD2.G packaging vectors. Lentivirus particles released from HEK293T cells were harvested and filtered and then used to infect MSCs. Puromycin was used to select cells expressing ATP9A-OV.

### 2.3. ATP9A Knockout

The lentiCRISPRv2 plasmid was used, as this vector expresses Cas9 nuclease and inserts ATP9A-specific single-guide RNA (sgRNA) simultaneously [19]. The online tool CRISPR DESIGN (https://CRISPR.mit.edu) was used to design sgRNA. The oligo sequence 5′-AGGAGATCCGATGCTACGTG-3′ was created and then annealed before subcloning into lentiCRISPRv2. To produce the lentivirus, cloned lenti-CRISPRv2 sgATP9A plasmids were transferred into HEK293T cells with packaging plasmids pMD2.G and psPAX2 using Lipofectamine 3000 (Invitrogen, Carlsbad, CA, USA). MSCs were infected with purified lentivirus, and a monoclonal cell line was selected using puromycin.

### 2.4. Confocal Microscopy

Cells were stably infected with lentivirus ATP9A-RFP on a 35 mm confocal dish at 37°C. After 48 h, cells were washed twice with PBS. To fix the cells, 4% paraformaldehyde was used. DAPI-containing mounting medium was used to mount the cells. Using a 60x oil objective, an LSM 800 confocal laser scanning microscope (Carl Zeiss) was used to collect images. The device was equipped with DAPI (405 nm) and red (568 nm) lasers.

### 2.5. Quantification of EVs in Culture Medium

To detect and analyze the concentrations and sizes of exosomes, NanoSight N3000 (Malvern, Ltd., Malvern, UK) nanoparticle tracking analysis (NTA) was used. The measuring duration was 60 s, and parameters were applied according to the manufacturer's instructions. The EVs were diluted to 1 ml with PBS; the NTA 3.2 software (version 3.2.16) calculated exosome distribution and size [20].

### 2.6. Purification of Exosomes

To remove apoptotic cell bodies and cellular debris, the medium (20 ml) was centrifuged at 200 G for 10 min, and then again at 2,000 G for 20 min. To remove macrovesicles, the supernatant was ultracentrifuged at 10,000 G for 25 min. Ultracentrifugation was used at 100,000 G for 60 min to retrieve exosomes. Exosome pellets were washed once with PBS, then centrifuged again at 100,000 G for 60 min. The resultant pellets were resuspended in 500 *μ*l PBS. A Bradford assay was conducted to determine the concentration of protein. Western blotting was performed to detect exosome marker CD9, tumor susceptibility gene 101 (TSG101), Alix, and endoplasmic reticulum (ER) marker calnexin.

### 2.7. Metabolite Extraction and Lipidomics

A solution of exosome pellets in chloroform/methanol/water (2/1/1, *v*/*v*/*v*) was vortexed for 1 min then centrifuged for 10 min at 3000 G. The organic phage was harvested and transferred to a fresh tube, and nitrogen was used to lyophilize the phage. Isopropanol/methanol (1/1, *v*/*v*) solution (400 *μ*l) was used to reconstitute dried metabolites. These were then vortexed and centrifuged at 10,000 G for 5 min at 4°C. LC-MS and a Kinetex C18 column (100 × 2.1 mm, 1.9 *μ*m) were used to analyze supernatant. The flow rate was set at 0.4 ml/min. Lipidomic assays in positive mode and negative mode were conducted following the protocol described in a previous report [21].

Lipid Search v 4.0.20 software (Thermo Fisher Scientific, USA) was used to process the raw data obtained from the LC-S analysis. Each sample's data was normalized to total area. The sample number, normalized peak intensities, and all variate data (including retention time (rt) and charge-to-mass ratio (*m*/*z*)) were uploaded to SIMCA-P+ 12.0 software (Umetrics, Umea, Sweden). Multivariate analyses were performed to categorize the lipid samples; this included orthogonal partial least squares discriminant analysis (OPLS-DA) and principal component analysis (PCA). A permutation test with the permutation number of 200 was used to validate the OPLS-DA models [22].

Using Lipid Search software (Thermo Fisher Scientific, USA), a qualitative analysis of the lipid metabolites was performed. The information collected included the type of lipids, chain length, and number of saturated bonds. The screening conditions for the potential lipid biomarkers were determined by the lipid metabolites in the OPLS-DA model having variable importance in the projection (VIP) scores > 1 and *P* values obtained from the *t* − test < 0.05.

### 2.8. Statistical Analysis

GraphPad Prism 7.0 was used to determine whether differences between groups were significantly different. For differences between two groups, an unpaired, two-sided Student's *t*-test was conducted, whereas, for three or more groups, a one-way ANOVA was used. Statistical significance is set as *P* < 0.05.

## 3. Results

**Figure 1:**
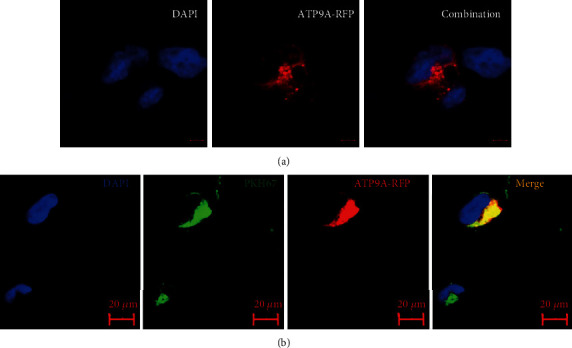
ATP9A localizes to intracellular vesicles and the plasma membrane. (a) To investigate ATP9A localization, HEK293 cells overexpressing ATP9A-RFP were detected using confocal microscopy. (b) HEK293 cells overexpressing ATP9A-RFP were labeled with general cell membrane dye PKH67-green.

To explore ATP9A's role in EV release in HEK293 cells, the location of ATP9A in the cells was examined. Using red fluorescent protein- (RFP-) tagged ATP9A (ATP9A-OV), it was noted that the location of ATP9A in the cells was accumulated primarily in intracellular vesicles (Figure 1).

**Figure 2:**
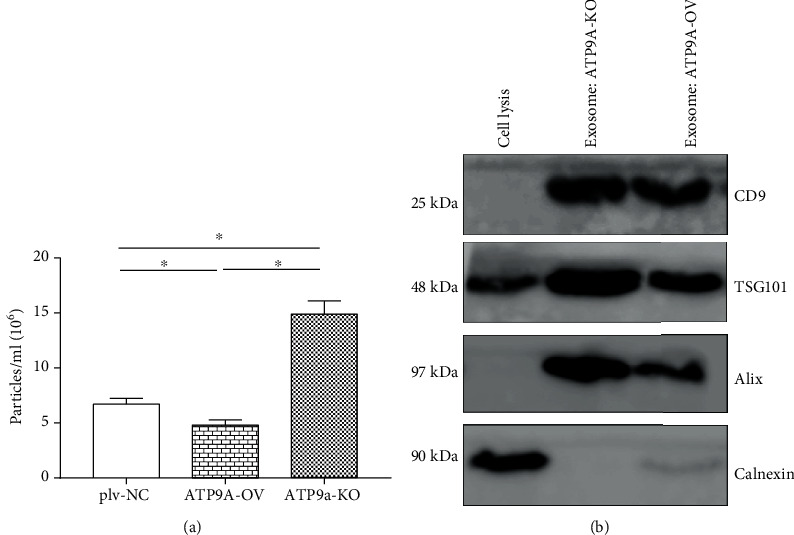
The total number of EVs is significantly greater in ATP9A knockdown cells. (a) EV secretion was evaluated in ATP9A knockdown (ATP9A-KO), ATP9A overexpression (ATP9A-OV), and plv-NC cells. (b) Exosome preparation was positive for exosome markers CD9, TSG101, and Alix and negative for ER marker calnexin. Tests were repeated in triplicate. ^∗^*P* < 0.05.

As ATP9A was detected at the plasma membrane and intracellular vesicles, reduced expression of ATP9A could affect ectosome or exosome release.  Figure 2 demonstrates that the number of EVs emitted in ATP9A-KO (ATP9A knockout cells) was significantly greater than the number of EVs released by the ATP9A-OV and plv-NC groups. The ATP9A-OV group released the fewest EVs. These findings suggest that the observed increase in the number of EVs from ATP9A knockout cells is mainly caused by elevated secretion of exosomes.

**Figure 3:**
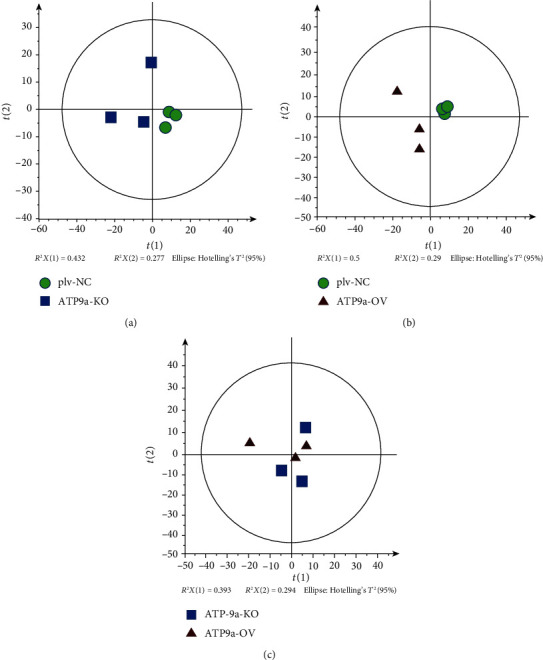
The PCA analysis of lipids in the plv-NC, ATP9A-OV, and ATP9A-KO groups. The PCA score plots obtained by comparing the three groups showed that three clusters were separated in the positive mode by the first two components. (a) PCA score between the plv-NC and ATP9A-KO groups. (b) PCA score between the plv-NC and ATP9A-OV groups. (c) PCA score between the ATP9A-KO and ATP9A-OV groups.

Lipidomics were performed for plv-NC, ATP9A-OV, and ATP9A-KO cells to better characterize the effects of ATP9A on exosome biosynthesis. Principal component analysis (PCA) showed that in the positive mode, the *R*^2^*X* and *Q*^2^ between the ATP9A-KO and plv-NC groups were 0.659 and -0.00879, respectively. Between the ATP9A-OV and plv-NC groups, the *R*^2^*X* and *Q*^2^ were 0.79 and 0.477, respectively. Finally, between the ATP9A-KO and ATP9A-OV groups, the *R*^2^*X* and *Q*^2^ values were 0.689 and 0.0479, respectively (Figure 3). As the *R*^2^*X* values all exceeded 0.4, the models were considered reliable. The PCA score plots obtained by comparing the three groups revealed that three clusters were separated in the positive mode by the first two components.

**Figure 4:**
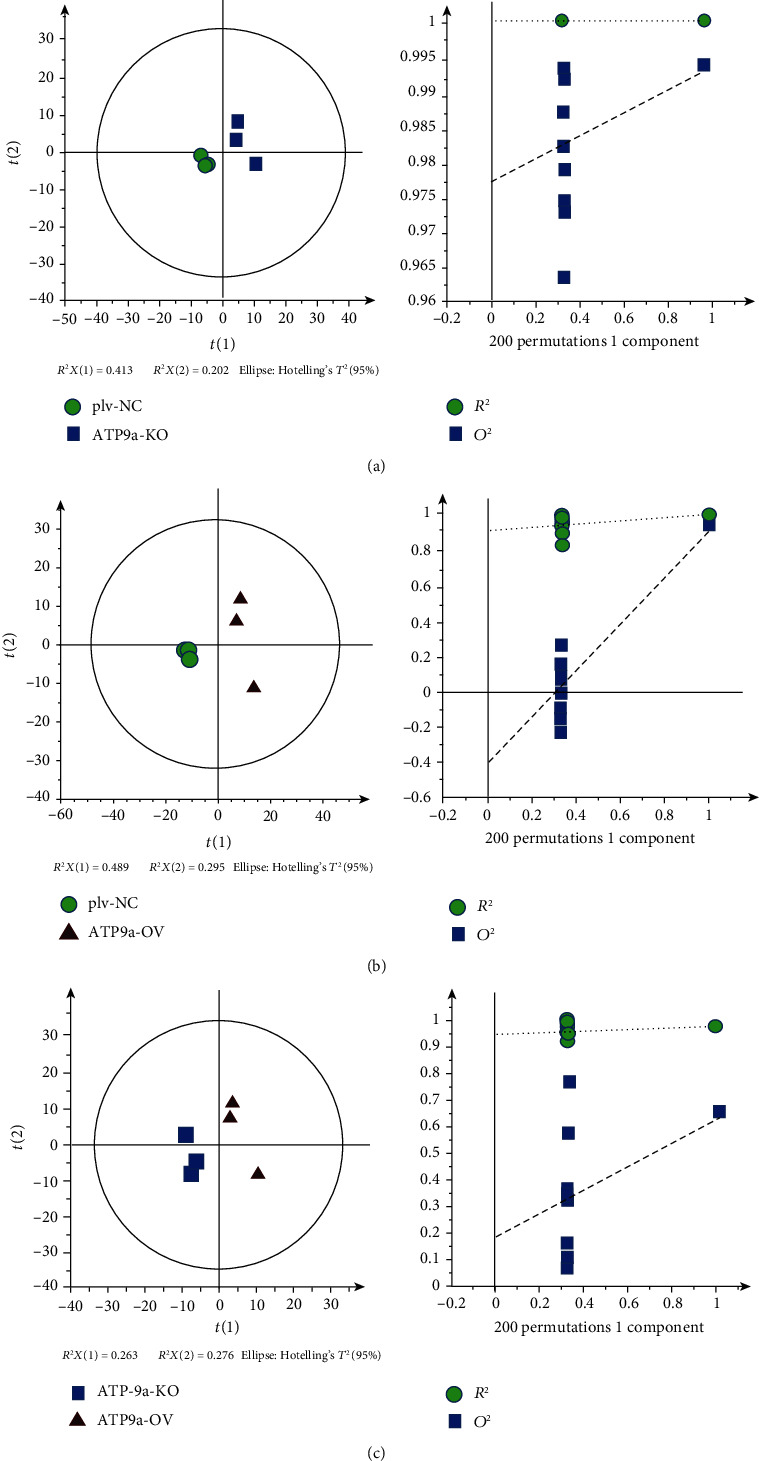
The OPLS-DA score plots of the plv-NC, ATP9A-OV, and ATP9A-KO groups. (a) OPLS-DA score between the plv-NC and ATP9A-KO groups. (b) OPLS-DA score between the plv-NC and ATP9A-OV groups. (c) OPLS-DA score between the ATP9A-KO and ATP9A-OV groups.

Figure 4 presents the PLS-DA score plots for the comparison of the three groups. This clearly shows separation in positive mode. The respective *R*^2^*X*, *R*^2^*Y*, and *Q*^2^ between the ATP9A-KO and plv-NC groups were 0.615, 0.983, and 0.851. Similarly, the respective *R*^2^*X*, *R*^2^*Y*, and *Q*^2^ values between the ATP9A-OV and plv-NC groups were 0.783, 0.99, and 0.951. Finally, the same respective values between the ATP9A-KO and ATP9A-OV groups were 0.539, 0.98, and 0.676.

**Figure 5:**
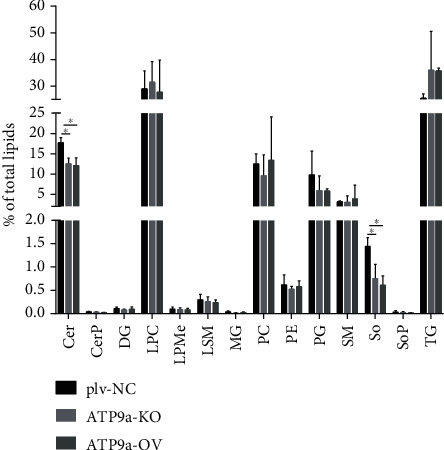
Percentage of all classes of lipids detected in the plv-NC, ATP9A-OV, and ATP9A-KO groups. Ceramides, lysophosphatidylcholine, phosphatidylglycerol, sphingomyelin, and triglycerides were the most common types of lipids. The percentage of ceramides (Cer), diacylglycerol (DG), lysophosphatidylcholine (LPC), lysophosphatidylmethanol (LPMe), monoglyceride (MG), phosphatidylcholine (PC), phosphatidylethanolamine (PE), phosphatidylglycerol (PG), sphingomyelin (SM), sphingoshine (So), and triglyceride (TG) are shown.

Figure 5 presents the amount of different lipids as a percentage of the total. The percentage of Cer was significantly greater in the plv-NC group than in the ATP9A-OV and ATP9A-KO groups (*P* < 0.05). The level of sphingosine (So) was greatest in the plv-NC group (*P* < 0.05).

In total, there was a difference of 7 lipid classes, comprising 49 significantly different lipid metabolites, between the ATP9A-KO and plv-NC groups. This difference increased to 8 lipid classes and 137 significantly different lipid metabolites between the ATP9A-OV and plv-NC groups. The ATP9A-KO and ATP9A-OV groups differed by 5 lipid classes and 10 significantly different lipid metabolites (Table 1). The detailed differences in lipid metabolites between the plv-NC, ATP9A-KO, and ATP9A-OV groups in the positive mode are shown in Tables 2, 3, and 4.  Table 5 shows the detailed differences in lipid metabolites between the ATP9A-OV and plv-NC groups in negative mode.

## 4. Discussion

ATP9A, a trafficking protein, plays a key role in the initiation of vesicle biogenesis [7, 23]. In this study, we found that the release of EVs from HEK293 cells was modulated by P4-ATPase ATP9A. The percentage of ceramides and sphingosine was significantly greater in the normal control cells than in the ATP9A overexpression and ATP9A knockout cells. The subcellular localization of mammalian P4-ATPases was observed using fluorescently labeled phospholipids to visualize the transport of phospholipids into cells. ATP9A was detected in the plasma membrane and intracellular compartments. This suggests that the presence of ATP9A in the plasma membrane is important for the purpose of assisting in the recycling of plasma membrane proteins through endocytosis [23]. EV release was increased in cell lines when ATP9A was knocked out; this implies that ATP9A plays an inhibitory role in EV release.

Many biological processes, such as angiogenesis, blood clotting, and immune response, are modulated by proteins, DNA, miRNA, and mRNAs, which could be controlled via EV-instigated intercellular communication. In this study, we demonstrated that exosome secretion was inhibited by ATP9A and this could be achieved through glucose transporter 1 (GLUT1) and transferrin receptor, as they are recycled to the plasma membrane through ATP9A endocytic recycling [17]. The knockdown expression of ATP9A in human hepatoma cells shows that the elevated release of EVs is independent of the activation of caspase-3 [23]. The quantity of EVs was significantly reduced in ATP9A knockdown cells in which exosome release had been pharmacologically blocked [23].

It is also possible that the elevated secretion of EVs is secondary to disrupting other key processes. ATP9A deficiency may be associated with cell death, proliferation, and survival pathways. Indeed, in HepG2 cells in which ATP9A was minimal, the cells underwent massive cell death approximately two weeks after ATP9A had been exhausted. As mentioned previously, ATP9A is a member of subclass 2 in P4-ATPases [11]; Neo1 (*Saccharomyces cerevisiae*) and TAT-5 (*C*. *elegans*) are orthologs of ATP9A. While it is recognized that P4-ATPase deficiency can result in lethal consequences, it is not yet known what molecular mechanisms are responsible for this phenomenon [24]. Hela cells containing CRISPR-mediated knockout of ATP9A did not die; however, it is noted that data for long-term culture were omitted [17]. This suggests that some cell types can withstand ATP9A depletion; the pathway in which the protein is involved may determine the outcome.

P4-ATPases are important to many plasma and intracellular membrane-associated processes. One of these enzymes' primary functions is to institute and maintain a state of phospholipid asymmetry of cell membranes [7]. Increased exposure of PS in the exoplasmic leaflet of ATP9A-depleted HepG2 cells resulted in partial loss of plasma membrane asymmetry [23]. In *S. cerevisiae*, the asymmetry of the plasma membrane is lost as PE and PS become exposed as a consequence of NEO1 deficiency [24]. Similarly, the exposure of PE in the exoplasmic leaflet of *C*. *elegans* increases with TAT-5 deficiency; however, no external PS was detected [16]. In this study, ATP9A-depleted HEK293 cells did not exhibit an increase in PS, but ceramides (Cer) and sphingosine were decreased in cells in which ATP9A was overexpressed or knocked out. A number of pathologies are associated with dysregulated sphingolipid metabolism; these include cancer, cardiovascular disease, hepatic steatosis, metabolic disorders, neurological disease, and type 2 diabetes [25, 26]. Exosomes were exchanged between the tumor cells and other tissues; however, few studies have examined the functional roles of these exosomal lipids [27]. Cer is enriched in the exosomes, and it is also an important lipid in exosomal formation. As the center in the sphingolipid metabolism, Cer is predominantly produced via acid sphingomyelinase-mediated hydrolysis of sphingomyelin and then metabolized to sphingosine by acid ceramidase [28]. Cosker and Segal suggested that the exosomal lipids can transmit “mobile rafts” that can activate cell signaling pathways in oncogenesis and metastasis [29]. Cer can modulate the roles of these mobile rafts and their effects on the signaling pathways [29]. Sphingosine may increase the activation of the TRPML1 channel in the presence of ML-SA1. ATP9A may regulate sphingosine production and, thus, decrease TRPML1 channel activity [30]. It was previously postulated that the translocation undertaken by P4-ATPases was selective, favoring glycerophospholipids and excluding sphingolipids. Thus, the molecular relationship between *ATP9A* and ceramides and sphingosine are mysterious [31].

## Figures and Tables

**Table 1:** The different lipid metabolites between the plv-NC, ATP9A-KO, and ATP9A-OV groups.

Lipid class	ATP9A-KO and plv-NC			ATP9A-OV and plv-NC			ATP9A-KO and ATP9A-OV		
	Positive mode	Negative mode	Total	Positive mode	Negative mode	Total	Positive mode	Negative mode	Total
Phosphatidylcholine (PC)	6	—	6	16	4	20	—	—	0
Phosphatidylethanolamine (PE)	2	—	2	3	—	3	2	—	2
Phosphatidylglycerol (PG)	1	—	1	—	—	0	—	—	0
Lysophosphatidylcholine (LPC)	8	—	8	12	1	13	4	—	4
Phosphatidylinositol (Pl)	—	—	0	—	—	0	2	—	2
Triglyceride (TG)	21	—	21	87	—	87	—	—	0
Diacylglycerol (DG)	—	—	0	1	—	1	1	—	1
Monogalactosylmonoacylglycerol (MGDG)	—	—	0	—	1	1	—	—	0
Sphingoshine (So)	1	—	1	2	—	2	—	—	0
Ceramides (Cer)	10	—	10	10	—	10	1	—	1
Total	49	—	49	131	6	137	10	—	10

**Table 2:** Detailed differences in lipid metabolites between the ATP9A-KO and plv-NC groups in positive mode.

No.	Lipid ion	Lipid group	Class	Fatty acid	Ion formula	VIP	*T*-test	Log_FC (ATP9a-KO/plv-NC)
1	Cer(d20 : 0/18 : 0) + H	Cer(d38 : 0) + H	Cer	(d20 : 0/18 : 0)	C38 H78 O3 N1	1.2604	0.030618969	-3.831098851
2	Cer(d22 : 0/18 : 0) + H	Cer(d40 : 0) + H	Cer	(d22 : 0/18 : 0)	C40 H82 O3 N1	1.24235	0.025143001	-4.328152705
3	Cer(d28 : 0) + H	Cer(d28 : 0) + H	Cer	(d28 : 0)	C28 H58 O3 N1	1.28102	0.0294742	-4.24959194
4	Cer(d30 : 0) + H	Cer(d30 : 0) + H	Cer	(d30 : 0)	C30 H62 O3 N1	1.275	0.025176996	-3.979850415
5	Cer(d32 : 0) + H	Cer(d32 : 0) + H	Cer	(d32 : 0)	C32 H66 O3 N1	1.26661	0.023622931	-4.490550701
6	Cer(d34 : 0) + H	Cer(d34 : 0) + H	Cer	(d34 : 0)	C34 H70 O3 N1	1.25672	0.029461839	-4.056379416
7	Cer(d34 : 0) + H	Cer(d34 : 0) + H	Cer	(d34 : 0)	C34 H70 O3 N1	1.23012	0.04426622	-3.117818449
8	Cer(d36 : 0) + H	Cer(d36 : 0) + H	Cer	(d36 : 0)	C36 H74 O3 N1	1.34994	0.008080528	-4.472595341
9	Cer(d36 : 3) + H	Cer(d36 : 3) + H	Cer	(d36 : 3)	C36 H68 O3 N1	1.29906	0.015827678	-4.504241793
10	Cer(d38 : 1) + H	Cer(d38 : 1) + H	Cer	(d38 : 1)	C38 H76 O3 N1	1.3665	0.004044369	-4.339760529
11	LPC(16 : 0) + H	LPC(16 : 0) + H	LPC	(16 : 0)	C24 H51 O7 N1 P1	1.19626	0.044904373	-3.965795361
12	LPC(16 : 0) + Na	LPC(16 : 0) + Na	LPC	(16 : 0)	C24 H50 O7 N1 P1 Na1	1.2719	0.019244897	-4.12685101
13	LPC(16 : 1) + H	LPC(16 : 1) + H	LPC	(16 : 1)	C24 H49 O7 N1 P1	1.37999	0.002239195	-4.666120577
14	LPC(18 : 1) + H	LPC(18 : 1) + H	LPC	(18 : 1)	C26 H53 O7 N1 P1	1.27108	0.019255048	-3.993299768
15	LPC(18 : 1) + H	LPC(18 : 1) + H	LPC	(18 : 1)	C26 H53 O7 N1 P1	1.27193	0.022622097	-5.420039555
16	LPC(18 : 3) + H	LPC(18 : 3) + H	LPC	(18 : 3)	C26 H49 O7 N1 P1	1.21783	0.033842529	-3.871958979
17	LPC(20 : 4) + H	LPC(20 : 4) + H	LPC	(20 : 4)	C28 H51 O7 N1 P1	1.3028	0.013961964	-5.206227559
18	LPC(22 : 6) + H	LPC(22 : 6) + H	LPC	(22 : 6)	C30 H51 O7 N1 P1	1.26602	0.021855354	-4.887480292
19	PC(31 : 0) + H	PC(31 : 0) + H	PC	(31 : 0)	C39 H79 O8 N1 P1	1.25034	0.043489185	-2.736580322
20	PC(33 : 0e) + H	PC(33 : 0e) + H	PC	(33 : 0e)	C41 H85 O7 N1 P1	1.24296	0.034649083	-4.296299639
21	PC(36 : 3) + H	PC(36 : 3) + H	PC	(36 : 3)	C44 H83 O8 N1 P1	1.25828	0.026138449	-2.824548745
22	PC(36 : 3) + H	PC(36 : 3) + H	PC	(36 : 3)	C44 H83 O8 N1 P1	1.22197	0.043015998	-2.834383398
23	PC(38 : 5) + H	PC(38 : 5) + H	PC	(38 : 5)	C46 H83 O8 N1 P1	1.26417	0.032636589	-2.865997476
24	PC(38 : 6) + H	PC(38 : 6) + H	PC	(38 : 6)	C46 H81 O8 N1 P1	1.2539	0.034802661	-2.826696561
25	PE(34 : 1e) + Na	PE(34 : 1e) + Na	PE	(34:1e)	C39 H78 O7 N1 P1 Na1	1.24296	0.034649083	-4.296299639
26	PE(38 : 6) + H	PE(38 : 6) + H	PE	(38 : 6)	C43 H75 O8 N1 P1	1.31751	0.016479104	-4.448539426
27	PG(45 : 6) + NH_4_	PG(45 : 6) + NH_4_	PG	(45 : 6)	C51 H93 O10 N1 P1	1.24296	0.034649083	-4.296299639
28	So(d16 : 0) + H	So(d16 : 0) + H	So	(d16 : 0)	C16 H36 O2 N1	1.25709	0.02502734	-2.937662223
29	TG(15 : 0/14 : 0/14 : 1) + NH_4_	TG(43 : 1) + NH_4_	TG	(15 : 0/14 : 0/14 : 1)	C46 H90 O6 N1	1.28754	0.026596333	-0.398603466
30	TG(15 : 0/17 : 0/17 : 0) + NH_4_	TG(49 : 0) + NH_4_	TG	(15 : 0/17 : 0/17 : 0)	C52 H104 O6 N1	1.3776	0.0031683	-2.264427759
31	TG(16 : 0/16 : 1/16 : 1) + NH_4_	TG(48 : 2) + NH_4_	TG	(16 : 0/16 : 1/16 : 1)	C51 H98 O6 N1	1.24966	0.034179356	-4.494068391
32	TG(16 : 0/18 : 1/18 : 1) + NH_4_	TG(52 : 2) + NH_4_	TG	(16 : 0/18 : 1/18 : 1)	C55 H106 O6 N1	1.27119	0.024617268	-2.876897982
33	TG(16 : 0/18 : 1/18 : 1) + NH_4_	TG(52 : 2) + NH_4_	TG	(16 : 0/18 : 1/18 : 1)	C55 H106 O6 N1	1.24273	0.029055366	-5.502551812
34	TG(16 : 1/16 : 1/16 : 1) + NH_4_	TG(48 : 3) + NH_4_	TG	(16 : 1/16 : 1/16 : 1)	C51 H96 O6 N1	1.25074	0.037153469	-2.820151763
35	TG(16 : 1/16 : 1/18 : 1) + Na	TG(50 : 3) + Na	TG	(16 : 1/16 : 1/18 : 1)	C53 H96 O6 Na1	1.38231	0.001565792	-4.888822805
36	TG(16 : 1/16 : 1/18 : 1) + NH_4_	TG(50 : 3) + NH_4_	TG	(16 : 1/16 : 1/18 : 1)	C53 H100 O6 N1	1.29488	0.013430777	-4.189332374
37	TG(16 : 1/18 : 1/18 : 1) + Na	TG(52 : 3) + Na	TG	(16 : 1/18 : 1/18 : 1)	C55 H100 O6 Na1	1.31766	0.013435952	-3.30410743
38	TG(16 : 1/18 : 1/18 : 1) + NH_4_	TG(52 : 3) + NH_4_	TG	(16 : 1/18 : 1/18 : 1)	C55 H104 O6 N1	1.30996	0.013226079	-3.528809232
39	TG(18 : 0/17 : 0/18 : 1) + NH_4_	TG(53 : 1) + NH_4_	TG	(18 : 0/17 : 0/18 : 1)	C56 H110 O6 N1	1.29598	0.022872573	0.90918934
40	TG(18 : 1/18 : 1/18 : 2) + NH_4_	TG(54 : 4) + NH_4_	TG	(18 : 1/18 : 1/18 : 2)	C57 H106 O6 N1	1.2432	0.044586752	-5.336667642
41	TG(18 : 1/18 : 2/18 : 2) + NH_4_	TG(54 : 5) + NH_4_	TG	(18 : 1/18 : 2/18 : 2)	C57 H104 O6 N1	1.2438	0.043561191	-2.874871415
42	TG(18 : 2/18 : 2/18 : 2) + Na	TG(54 : 6) + Na	TG	(18 : 2/18 : 2/18 : 2)	C57 H98 O6 Na1	1.28903	0.026337165	-4.660675027
43	TG(18 : 2/18 : 2/18 : 2) + NH_4_	TG(54 : 6) + NH_4_	TG	(18 : 2/18 : 2/18 : 2)	C57 H102 O6 N1	1.22084	0.039362783	-4.36954712
44	TG(18 : 4/18 : 1/18 : 1) + H	TG(54 : 6) + H	TG	(18 : 4/18 : 1/18 : 1)	C57 H99 O6	1.21603	0.04656314	-3.727194618
45	TG(20 : 5/18 : 2/18 : 2) + H	TG(56 : 9) + H	TG	(20 : 5/18 : 2/18 : 2)	C59 H97 O6	1.34412	0.00940743	-5.064000583
46	TG(4 : 0/16 : 0/18 : 0) + NH_4_	TG(38 : 0) + NH_4_	TG	(4 : 0/16 : 0/18 : 0)	C41 H82 O6 N1	1.30211	0.020072444	-0.008648167
47	TG(4 : 0/18 : 0/18 : 0) + NH_4_	TG(40 : 0) + NH_4_	TG	(4 : 0/18 : 0/18 : 0)	C43 H86 O6 N1	1.39232	0.001247294	-1.058028038
48	TG(6 : 0/16 : 0/20 : 4) + H	TG(42 : 4) + H	TG	(6 : 0/16 : 0/20 : 4)	C45 H79 O6	1.36281	0.005419013	4.422086595
49	TG(6 : 0/18 : 1/18 : 1) + NH_4_	TG(42 : 2) + NH_4_	TG	(6 : 0/18 : 1/18 : 1)	C45 H86 O6 N1	1.24028	0.047259192	18.15651265

**Table 3:** Detailed differences in lipid metabolites between the ATP9A-KO and plv-NC groups in positive mode.

No.	Lipid ion	Lipid group	Class	Fatty acid	Ion formula	VIP	*T*-test	Log_FC(ATP9a-OV/plv-NC)
1	Cer(d20 : 0/18 : 0) + H	Cer(d38 : 0) + H	Cer	(d20 : 0/18 : 0)	C38 H78 O3 N1	1.18056	0.011353875	-0.412362667
2	Cer(d22 : 0/18 : 0) + H	Cer(d40 : 0) + H	Cer	(d22 : 0/18 : 0)	C40 H82 O3 N1	1.15478	0.017086722	-0.595999589
3	Cer(d22 : 1/2 : 0) + H	Cer(d24 : 1) + H	Cer	(d22 : 1/2 : 0)	C24 H48 O3 N1	1.11826	0.043499783	-0.570814243
4	Cer(d30 : 0) + H	Cer(d30 : 0) + H	Cer	(d30 : 0)	C30 H62 O3 N1	1.15035	0.025673707	-0.586615549
5	Cer(d32 : 0) + H	Cer(d32 : 0) + H	Cer	(d32 : 0)	C32 H66 O3 N1	1.10775	0.043725597	-0.631568883
6	Cer(d32 : 0) + H	Cer(d32 : 0) + H	Cer	(d32 : 0)	C32 H66 O3 N1	1.11214	0.048398875	-0.841046781
7	Cer(d32 : 0) + H	Cer(d32 : 0) + H	Cer	(d32 : 0)	C32 H66 O3 N1	1.13423	0.041166313	-0.537395468
8	Cer(d34 : 0) + H	Cer(d34 : 0) + H	Cer	(d34 : 0)	C34 H70 O3 N1	1.15175	0.021192786	-0.739156196
9	Cer(d36 : 0) + H	Cer(d36 : 0) + H	Cer	(d36 : 0)	C36 H74 O3 N1	1.1546	0.028080187	-0.343491384
10	Cer(d38 : 1) + H	Cer(d38 : 1) + H	Cer	(d38 : 1)	C38 H76 O3 N1	1.25148	0.000869044	-0.708510594
11	DG(18 : 1/18 : 1) + NH_4_	DG(36 : 2) + NH_4_	DG	(18 : 1/18 : 1)	C39 H76 O5 N1	1.14775	0.032034195	-1.170941291
12	LPC(14 : 0) + H	LPC(14 : 0) + H	LPC	(14 : 0)	C22 H47 O7 N1 P1	1.17492	0.011401697	-2.037925913
13	LPC(16 : 0) + H	LPC(16 : 0) + H	LPC	(16 : 0)	C24 H51 O7 N1 P1	1.18361	0.015200139	-1.185399006
14	LPC(16 : 0) + H	LPC(16 : 0) + H	LPC	(16 : 0)	C24 H51 O7 N1 P1	1.16722	0.016014207	-1.586333123
15	LPC(16 : 0) + Na	LPC(16 : 0) + Na	LPC	(16 : 0)	C24 H50 O7 N1 P1 Na1	1.2007	0.006996003	-1.69267157
16	LPC(16 : 1) + H	LPC(16 : 1) + H	LPC	(16 : 1)	C24 H49 O7 N1 P1	1.25501	0.000734215	-1.973182318
17	LPC(18 : 0) + H	LPC(18 : 0) + H	LPC	(18 : 0)	C26 H55 O7 N1 P1	1.17629	0.009004989	-1.955935386
18	LPC(18 : 1) + H	LPC(18 : 1) + H	LPC	(18 : 1)	C26 H53 O7 N1 P1	1.19239	0.008904148	-1.722036886
19	LPC(18 : 1) + H	LPC(18 : 1) + H	LPC	(18 : 1)	C26 H53 O7 N1 P1	1.17863	0.013931838	-1.295055719
20	LPC(18 : 2) + H	LPC(18 : 2) + H	LPC	(18 : 2)	C26 H51 O7 N1 P1	1.09975	0.033368835	-1.836675727
21	LPC(18 : 3) + H	LPC(18 : 3) + H	LPC	(18 : 3)	C26 H49 O7 N1 P1	1.17536	0.011863232	-1.692803017
22	LPC(20 : 4) + H	LPC(20 : 4) + H	LPC	(20 : 4)	C28 H51 O7 N1 P1	1.20989	0.006643335	-1.601519506
23	LPC(22 : 6) + H	LPC(22 : 6) + H	LPC	(22 : 6)	C30 H51 O7 N1 P1	1.18109	0.012416167	-1.660861569
24	PC(16 : 0/20 : 4) + Na	PC(36 : 4) + Na	PC	(16 : 0/20 : 4)	C44 H80 O8 N1 P1 Na1	1.18728	0.009695918	-1.197287937
25	PC(18 : 0/20 : 4) + Na	PC(38 : 4) + Na	PC	(18 : 0/20 : 4)	C46 H84 O8 N1 P1 Na1	1.19373	0.01387346	-1.252308071
26	PC(34 : 2) + H	PC(34 : 2) + H	PC	(34 : 2)	C42 H81 O8 N1 P1	1.13145	0.021697615	-1.645003452
27	PC(34 : 2) + Na	PC(34 : 2) + Na	PC	(34 : 2)	C42 H80 O8 N1 P1 Na1	1.14273	0.022076089	-2.192884674
28	PC(36 : 2) + H	PC(36 : 2) + H	PC	(36 : 2)	C44 H85 O8 N1 P1	1.18637	0.009290191	-1.520413569
29	PC(36 : 3) + H	PC(36 : 3) + H	PC	(36 : 3)	C44 H83 O8 N1 P1	1.2034	0.00592586	-1.534810921
30	PC(36 : 3) + H	PC(36 : 3) + H	PC	(36 : 3)	C44 H83 O8 N1 P1	1.091	0.063303852	-0.584433881
31	PC(36 : 3) + H	PC(36 : 3) + H	PC	(36 : 3)	C44 H83 O8 N1 P1	1.17719	0.013773598	-1.530770278
32	PC(36 : 4) + H	PC(36 : 4) + H	PC	(36 : 4)	C44 H81 O8 N1 P1	1.15291	0.019974882	-1.409087872
33	PC(36 : 4) + H	PC(36 : 4) + H	PC	(16 : 0/20 : 4)	C44 H81 O8 N1 P1	1.15056	0.024222573	-1.151908928
34	PC(36 : 4p) + H	PC(36 : 4p) + H	PC	(36 : 4p)	C44 H81 O7 N1 P1	1.09667	0.056620135	-1.032394903
35	PC(38 : 4) + H	PC(38 : 4) + H	PC	(18 : 0/20 : 4)	C46 H85 O8 N1 P1	1.01835	0.138748412	-0.52148975
36	PC(38 : 5) + H	PC(38 : 5) + H	PC	(38 : 5)	C46 H83 O8 N1 P1	1.10915	0.058086289	-0.647388207
37	PC(38 : 6) + H	PC(38 : 6) + H	PC	(38 : 6)	C46 H81 O8 N1 P1	1.22093	0.004912756	-1.089933633
38	PC(38 : 6) + Na	PC(38 : 6) + Na	PC	(38 : 6)	C46 H80 O8 N1 P1 Na1	1.16989	0.018637803	-1.222573329
39	PC(40 : 7) + H	PC(40 : 7) + H	PC	(40 : 7)	C48 H83 O8 N1 P1	1.11113	0.053579975	-0.945260739
40	PE(16 : 0p/18 : 1) + H	PE(34 : 1p) + H	PE	(16 : 0p/18 : 1)	C39 H77 O7 N1 P1	1.11038	0.055327279	0.456373702
41	PE(38 : 6) + H	PE(38 : 6) + H	PE	(38 : 6)	C43 H75 O8 N1 P1	1.22639	0.004954273	-2.282070421
42	PE(39 : 5) + H	PE(39 : 5) + H	PE	(39 : 5)	C44 H79 O8 N1 P1	1.1443	0.017276536	-1.751279845
43	So(d14 : 1) + H	So(d14:1) + H	So	(d14 : 1)	C14 H30 O2 N1	1.17166	0.01993559	-2.081806693
44	So(d16 : 0) + H	So(d16 : 0) + H	So	(d16 : 0)	C16 H36 O2 N1	1.09733	0.046617899	-1.916789565
45	TG(12 : 0/12 : 0/18 : 3) + H	TG(42 : 3) + H	TG	(12 : 0/12 : 0/18 : 3)	C45 H81 O6	1.2558	0.000676947	1.943382457
46	TG(15 : 0/14 : 0/14 : 1) + NH_4_	TG(43 : 1) + NH_4_	TG	(15 : 0/14 : 0/14 : 1)	C46 H90 O6 N1	1.19099	0.014229158	1.640598605
47	TG(15 : 0/16 : 0/16 : 0) + Na	TG(47 : 0) + Na	TG	(15 : 0/16 : 0/16 : 0)	C50 H96 O6 Na1	1.1358	0.02142449	1.159764763
48	TG(15 : 0/16 : 0/16 : 1) + NH_4_	TG(47 : 1) + NH_4_	TG	(15 : 0/16 : 0/16 : 1)	C50 H98 O6 N1	1.03827	0.099079456	-0.913642961
49	TG(15 : 0/16 : 0/18 : 1) + NH_4_	TG(49 : 1) + NH_4_	TG	(15 : 0/16 : 0/18 : 1)	C52 H102 O6 N1	1.17672	0.017013743	0.731184335
50	TG(15 : 0/17 : 0/17 : 0) + NH_4_	TG(49 : 0) + NH_4_	TG	(15 : 0/17 : 0/17 : 0)	C52 H104 O6 N1	1.16807	0.022219937	1.500799668
51	TG(15 : 1/14 : 0/16 : 1) + NH_4_	TG(45 : 2) + NH_4_	TG	(15 : 1/14 : 0/16 : 1)	C48 H92 O6 N1	1.12638	0.046410662	1.228411695
52	TG(16 : 0/10 : 0/18 : 1) + NH_4_	TG(44 : 1) + NH_4_	TG	(16 : 0/10 : 0/18 : 1)	C47 H92 O6 N1	1.21473	0.006570563	1.270938382
53	TG(16 : 0/12 : 0/13 : 0) + NH_4_	TG(41 : 0) + NH_4_	TG	(16 : 0/12 : 0/13 : 0)	C44 H88 O6 N1	1.21837	0.006165409	1.937202801
54	TG(16 : 0/12 : 0/14 : 0) + Na	TG(42 : 0) + Na	TG	(16 : 0/12 : 0/14 : 0)	C45 H86 O6 Na1	1.20558	0.00933199	1.253529015
55	TG(16 : 0/12 : 0/14 : 0) + NH_4_	TG(42 : 0) + NH_4_	TG	(16 : 0/12 : 0/14 : 0)	C45 H90 O6 N1	1.12469	0.041448684	0.995570056
56	TG(16 : 0/12 : 0/18 : 1) + NH_4_	TG(46 : 1) + NH_4_	TG	(16 : 0/12 : 0/18 : 1)	C49 H96 O6 N1	1.21927	0.006268795	0.851464834
57	TG(16 : 0/13 : 0/14 : 0) + NH_4_	TG(43 : 0) + NH_4_	TG	(16 : 0/13 : 0/14 : 0)	C46 H92 O6 N1	1.11757	0.045489213	1.347749132
58	TG(16 : 0/14 : 0/14 : 0) + Na	TG(44 : 0) + Na	TG	(16 : 0/14 : 0/14 : 0)	C47 H90 O6 Na1	1.24805	0.001498992	1.376050197
59	TG(16 : 0/14 : 0/14 : 0) + NH_4_	TG(44 : 0) + NH_4_	TG	(16 : 0/14 : 0/14 : 0)	C47 H94 O6 N1	1.26237	0.000353143	0.962131291
60	TG(16 : 0/14 : 0/16 : 0) + Na	TG(46 : 0) + Na	TG	(16 : 0/14 : 0/16 : 0)	C49 H94 O6 Na1	1.26857	8.80516E-05	1.565797115
61	TG(16 : 0/14 : 0/16 : 0) + NH_4_	TG(46 : 0) + NH_4_	TG	(16 : 0/14 : 0/16 : 0)	C49 H98 O6 N1	1.21901	0.00635889	1.450016601
62	TG(16 : 0/14 : 0/20 : 4) + H	TG(50 : 4) + H	TG	(16 : 0/14 : 0/20 : 4)	C53 H95 O6	1.26854	9.83824E-05	1.326744372
63	TG(16 : 0/16 : 0/16 : 0) + Na	TG(48 : 0) + Na	TG	(16 : 0/16 : 0/16 : 0)	C51 H98 O6 Na1	1.26708	0.000130539	1.471325066
64	TG(16 : 0/16 : 0/16 : 0) + NH_4_	TG(48 : 0) + NH_4_	TG	(16 : 0/16 : 0/16 : 0)	C51 H102 O6 N1	1.25972	0.000420793	1.491554693
65	TG(16 : 0/16 : 0/16 : 1) + Na	TG(48 : 1) + Na	TG	(16 : 0/16 : 0/16 : 1)	C51 H96 O6 Na1	1.26997	6.14983E-05	1.144805299
66	TG(16 : 0/16 : 0/16 : 1) + NH_4_	TG(48 : 1) + NH_4_	TG	(16 : 0/16 : 0/16 : 1)	C51 H100 O6 N1	1.25463	0.000897787	1.053045297
67	TG(16 : 0/16 : 0/18 : 1) + Na	TG(50 : 1) + Na	TG	(16 : 0/16 : 0/18 : 1)	C53 H100 O6 Na1	1.25028	0.00106583	1.831200518
68	TG(16 : 0/16 : 0/18 : 1) + NH_4_	TG(50 : 1) + NH_4_	TG	(16 : 0/16 : 0/18 : 1)	C53 H104 O6 N1	1.25192	0.000771575	1.773752236
69	TG(16 : 0/16 : 0/20 : 3) + H	TG(52 : 3) + H	TG	(16 : 0/16 : 0/20 : 3)	C55 H101 O6	1.26734	0.000120746	2.280590338
70	TG(16 : 0/16 : 0/20 : 4) + H	TG(52 : 4) + H	TG	(16 : 0/16 : 0/20 : 4)	C55 H99 O6	1.25001	0.001163477	1.849719348
71	TG(16 : 0/16 : 1/18 : 1) + Na	TG(50 : 2) + Na	TG	(16 : 0/16 : 1/18 : 1)	C53 H98 O6 Na1	1.03902	0.051199679	-0.248516244
72	TG(16 : 0/17 : 0/18 : 1) + NH_4_	TG(51 : 1) + NH_4_	TG	(16 : 0/17 : 0/18 : 1)	C54 H106 O6 N1	1.12884	0.042802734	2.648612904
73	TG(16 : 0/18 : 1/18 : 1) + NH_4_	TG(52 : 2) + NH_4_	TG	(16 : 0/18 : 1/18 : 1)	C55 H106 O6 N1	1.04551	0.102202776	-1.220332232
74	TG(16 : 0/18 : 2/18 : 2) + Na	TG(52 : 4) + Na	TG	(16 : 0/18 : 2/18 : 2)	C55 H98 O6 Na1	1.01276	0.072197269	-0.233717192
75	TG(16 : 0/18 : 2/18 : 2) + NH_4_	TG(52 : 4) + NH_4_	TG	(16 : 0/18 : 2/18 : 2)	C55 H102 O6 N1	1.1579	0.015560565	-0.777716804
76	TG(16 : 0/8 : 0/18 : 1) + NH_4_	TG(42 : 1) + NH_4_	TG	(16 : 0/8 : 0/18 : 1)	C45 H88 O6 N1	1.27372	5.23657E-06	2.89136082
77	TG(16 : 1/16 : 1/18 : 1) + Na	TG(50 : 3) + Na	TG	(16 : 1/16 : 1/18 : 1)	C53 H96 O6 Na1	1.24888	0.000976709	-0.791307191
78	TG(16 : 1/16 : 1/18 : 1) + NH_4_	TG(50 : 3) + NH_4_	TG	(16 : 1/16 : 1/18 : 1)	C53 H100 O6 N1	1.16996	0.013073421	-0.641262818
79	TG(16 : 1/18 : 1/18 : 1) + Na	TG(52 : 3) + Na	TG	(16 : 1/18 : 1/18 : 1)	C55 H100 O6 Na1	1.22748	0.002533394	-0.763755159
80	TG(16 : 1/18 : 1/18 : 1) + NH_4_	TG(52 : 3) + NH_4_	TG	(16 : 1/18 : 1/18 : 1)	C55 H104 O6 N1	1.20162	0.005474227	-0.786386257
81	TG(18 : 0/16 : 0/16 : 0) + Na	TG(50 : 0) + Na	TG	(18 : 0/16 : 0/16 : 0)	C53 H102 O6 Na1	1.26614	0.000158837	2.275169817
82	TG(18 : 0/16 : 0/16 : 0) + NH_4_	TG(50 : 0) + NH_4_	TG	(18 : 0/16 : 0/16 : 0)	C53 H106 O6 N1	1.26293	0.000281314	2.032336625
83	TG(18 : 0/16 : 0/18 : 0) + Na	TG(52 : 0) + Na	TG	(18 : 0/16 : 0/18 : 0)	C55 H106 O6 Na1	1.25922	0.000520621	2.41337606
84	TG(18 : 0/16 : 0/18 : 0) + NH_4_	TG(52 : 0) + NH_4_	TG	(18 : 0/16 : 0/18 : 0)	C55 H110 O6 N1	1.26597	0.000180915	2.667777604
85	TG(18 : 0/16 : 0/18 : 1) + Na	TG(52 : 1) + Na	TG	(18 : 0/16 : 0/18 : 1)	C55 H104 O6 Na1	1.26724	0.000138532	2.887231449
86	TG(18 : 0/16 : 0/18 : 1) + NH_4_	TG(52 : 1) + NH_4_	TG	(18 : 0/16 : 0/18 : 1)	C55 H108 O6 N1	1.26045	0.000462091	3.231733656
87	TG(18 : 0/16 : 0/20 : 4) + H	TG(54 : 4) + H	TG	(18 : 0/16 : 0/20 : 4)	C57 H103 O6	1.26769	0.000124179	3.110340992
88	TG(18 : 0/16 : 0/22 : 0) + NH_4_	TG(56 : 0) + NH_4_	TG	(18 : 0/16 : 0/22 : 0)	C59 H118 O6 N1	1.22968	0.004316666	2.187805394
89	TG(18 : 0/17 : 0/18 : 0) + NH_4_	TG(53 : 0) + NH_4_	TG	(18 : 0/17 : 0/18 : 0)	C56 H112 O6 N1	1.26138	0.000396904	2.424298519
90	TG(18 : 0/17 : 0/18 : 1) + NH_4_	TG(53 : 1) + NH_4_	TG	(18 : 0/17 : 0/18 : 1)	C56 H110 O6 N1	1.18602	0.016664437	2.713496047
91	TG(18 : 0/18 : 0/18 : 0) + Na	TG(54 : 0) + Na	TG	(18 : 0/18 : 0/18 : 0)	C57 H110 O6 Na1	1.26827	0.000105223	5.230250311
92	TG(18 : 0/18 : 0/18 : 0) + NH_4_	TG(54 : 0) + NH_4_	TG	(18 : 0/18 : 0/18 : 0)	C57 H114 O6 N1	1.26949	5.7205E-05	2.285459979
93	TG(18 : 0/18 : 0/18 : 1) + Na	TG(54 : 1) + Na	TG	(18 : 0/18 : 0/18 : 1)	C57 H108 O6 Na1	1.26278	0.000305486	4.481228399
94	TG(18 : 0/18 : 0/18 : 1) + NH_4_	TG(54 : 1) + NH_4_	TG	(18 : 0/18 : 0/18 : 1)	C57 H112 O6 N1	1.27143	3.13706E-05	4.649314869
95	TG(18 : 0/18 : 0/18 : 3) + H	TG(54 : 3) + H	TG	(18 : 0/18 : 0/18 : 3)	C57 H105 O6	1.26198	0.000356975	2.416341259
96	TG(18 : 0/18 : 0/20 : 4) + H	TG(56 : 4) + H	TG	(18 : 0/18 : 0/20 : 4)	C59 H107 O6	1.26273	0.000307805	4.483895814
97	TG(18 : 0/18 : 1/18 : 1) + Na	TG(54 : 2) + Na	TG	(18 : 0/18 : 1/18 : 1)	C57 H106 O6 Na1	1.22634	0.002728095	1.662501
98	TG(18 : 0/18 : 1/18 : 1) + NH_4_	TG(54 : 2) + NH_4_	TG	(18 : 0/18 : 1/18 : 1)	C57 H110 O6 N1	1.19634	0.010041025	1.513228258
99	TG(18 : 1/18 : 1/18 : 2) + NH_4_	TG(54 : 4) + NH_4_	TG	(18 : 1/18 : 1/18 : 2)	C57 H106 O6 N1	1.15588	0.023234959	-0.351349117
100	TG(18 : 1/18 : 1/20 : 4) + H	TG(56 : 6) + H	TG	(18 : 1/18 : 1/20 : 4)	C59 H103 O6	1.04017	0.05964527	-0.182813869
101	TG(18 : 1/18 : 2/18 : 2) + NH_4_	TG(54 : 5) + NH_4_	TG	(18 : 1/18 : 2/18 : 2)	C57 H104 O6 N1	1.22517	0.004865563	-0.471280945
102	TG(18 : 2/18 : 2/18 : 2) + Na	TG(54 : 6) + Na	TG	(18 : 2/18 : 2/18 : 2)	C57 H98 O6 Na1	1.01999	0.138715238	-0.381333699
103	TG(18 : 2/18 : 2/18 : 2) + NH_4_	TG(54 : 6) + NH_4_	TG	(18 : 2/18 : 2/18 : 2)	C57 H102 O6 N1	1.14238	0.030646745	-0.792226116
104	TG(18 : 3/18 : 2/18 : 2) + H	TG(54 : 7) + H	TG	(18 : 3/18 : 2/18 : 2)	C57 H97 O6	1.0853	0.053095571	-0.233717192
105	TG(18 : 3/18 : 2/18 : 2) + NH_4_	TG(54 : 7) + NH_4_	TG	(18 : 3/18 : 2/18 : 2)	C57 H100 O6 N1	1.04634	0.083166907	-0.934006022
106	TG(18 : 4/18 : 1/18 : 1) + H	TG(54 : 6) + H	TG	(18 : 4/18 : 1/18 : 1)	C57 H99 O6	1.17609	0.009589781	-0.65252347
107	TG(20 : 0e/18 : 2/18 : 3) + H	TG(56 : 5e) + H	TG	(20 : 0e/18 : 2/18 : 3)	C59 H107 O5	1.20462	0.010060286	3.325380766
108	TG(20 : 5/18 : 2/18 : 2) + H	TG(56 : 9) + H	TG	(20 : 5/18 : 2/18 : 2)	C59 H97 O6	1.01499	0.144587695	-0.440537678
109	TG(4 : 0/12 : 0/18 : 1) + NH_4_	TG(34 : 1) + NH_4_	TG	(4 : 0/12 : 0/18 : 1)	C37 H72 O6 N1	1.20296	0.01036702	4.04828539
110	TG(4 : 0/12 : 0/18 : 3) + H	TG(34 : 3) + H	TG	(4 : 0/12 : 0/18 : 3)	C37 H65 O6	1.06475	0.062064921	0.316304726
111	TG(4 : 0/14 : 0/18 : 1) + NH_4_	TG(36 : 1) + NH_4_	TG	(4 : 0/14 : 0/18 : 1)	C39 H76 O6 N1	1.24976	0.001350972	23.88325332
112	TG(4 : 0/14 : 0/18 : 3) + H	TG(36 : 3) + H	TG	(4 : 0/14 : 0/18 : 3)	C39 H69 O6	1.14385	0.035066923	2.573454618
113	TG(4 : 0/15 : 0/16 : 0) + NH_4_	TG(35 : 0) + NH_4_	TG	(4 : 0/15 : 0/16 : 0)	C38 H76 O6 N1	1.26752	0.000121981	5.017537183
114	TG(4 : 0/15 : 0/18 : 1) + NH_4_	TG(37 : 1) + NH_4_	TG	(4 : 0/15 : 0/18 : 1)	C40 H78 O6 N1	1.2024	0.010822476	3.978439339
115	TG(4 : 0/16 : 0/17 : 0) + NH_4_	TG(37 : 0) + NH_4_	TG	(4 : 0/16 : 0/17 : 0)	C40 H80 O6 N1	1.26052	0.000457105	3.907668532
116	TG(4 : 0/16 : 0/18 : 0) + Na	TG(38 : 0) + Na	TG	(4 : 0/16 : 0/18 : 0)	C41 H78 O6 Na1	1.22345	0.005085456	2.38625847
117	TG(4 : 0/16 : 0/18 : 0) + NH_4_	TG(38 : 0) + NH_4_	TG	(4 : 0/16 : 0/18 : 0)	C41 H82 O6 N1	1.10835	0.058570888	1.040201462
118	TG(4 : 0/16 : 0/18 : 1) + Na	TG(38 : 1) + Na	TG	(4 : 0/16 : 0/18 : 1)	C41 H76 O6 Na1	1.25375	0.000949516	6.971333525
119	TG(4 : 0/16 : 0/18 : 1) + NH_4_	TG(38 : 1) + NH_4_	TG	(4 : 0/16 : 0/18 : 1)	C41 H80 O6 N1	1.25057	0.001235431	6.528128518
120	TG(4 : 0/16 : 0/20 : 3) + H	TG(40 : 3) + H	TG	(4 : 0/16 : 0/20 : 3)	C43 H77 O6	1.23489	0.003249599	3.288156904
121	TG(4 : 0/17 : 0/18 : 1) + NH_4_	TG(39 : 1) + NH_4_	TG	(4 : 0/17 : 0/18 : 1)	C42 H82 O6 N1	1.2527	0.001062263	4.151115681
122	TG(4 : 0/18 : 0/18 : 0) + NH_4_	TG(40 : 0) + NH_4_	TG	(4 : 0/18 : 0/18 : 0)	C43 H86 O6 N1	1.13529	0.036323412	1.926228297
123	TG(4 : 0/18 : 1/18 : 1) + Na	TG(40 : 2) + Na	TG	(4 : 0/18 : 1/18 : 1)	C43 H78 O6 Na1	1.22707	0.004439594	1.831811125
124	TG(4 : 0/18 : 1/18 : 1) + NH_4_	TG(40 : 2) + NH_4_	TG	(4 : 0/18 : 1/18 : 1)	C43 H82 O6 N1	1.22764	0.004258607	1.648333529
125	TG(4 : 0/18 : 1/18 : 3) + H	TG(40 : 4) + H	TG	(4 : 0/18 : 1/18 : 3)	C43 H75 O6	1.25375	0.000949516	6.971333525
126	TG(46 : 1) + Na	TG(46 : 1) + Na	TG	(16 : 0/12 : 0/18 : 1)	C49 H92 O6 Na1	1.23901	0.002745257	0.902280507
127	TG(6 : 0/16 : 0/17 : 0) + NH_4_	TG(39 : 0) + NH_4_	TG	(6 : 0/16 : 0/17 : 0)	C42 H84 O6 N1	1.25624	0.000728265	2.333598845
128	TG(6 : 0/16 : 0/18 : 1) + NH_4_	TG(40 : 1) + NH_4_	TG	(6 : 0/16 : 0/18 : 1)	C43 H84 O6 N1	1.26874	8.63677E-05	4.581376753
129	TG(6 : 0/16 : 0/20 : 4) + H	TG(42 : 4) + H	TG	(6 : 0/16 : 0/20 : 4)	C45 H79 O6	1.26166	0.000379843	7.405154525
130	TG(6 : 0/18 : 1/18 : 1) + NH_4_	TG(42 : 2) + NH_4_	TG	(6 : 0/18 : 1/18 : 1)	C45 H86 O6 N1	1.24437	0.001939902	19.65297904
131	TG(8 : 0/12 : 0/18 : 3) + H	TG(38 : 3) + H	TG	(8 : 0/12 : 0/18 : 3)	C41 H73 O6	1.19976	0.011885245	2.272770506

**Table 4:** Detailed differences in lipid metabolites between the ATP9A-OV and ATP9A-KO groups in positive mode.

No.	Lipid ion	Lipid group	Class	Fatty acid	Ion formula	VIP	*T*-test	Log_FC(ATP9a-OV/ATP9a-KO)
1	Cer(d32 : 0) + H	Cer(d32 : 0) + H	Cer	(d32 : 0)	C32 H66 O3 N1	1.53407	0.046202984	-1.221196739
2	DG(18 : 1/18 : 1) + NH_4_	DG(36 : 2) + NH_4_	DG	(18 : 1/18 : 1)	C39 H76 O5 N1	1.57489	0.033527384	-0.844934869
3	LPC(16 : 0) + H	LPC(16 : 0) + H	LPC	(16 : 0)	C24 H51 O7 N1 P1	1.55693	0.038858829	-0.646114065
4	LPC(16 : 1) + H	LPC(16 : 1) + H	LPC	(16 : 1)	C24 H49 O7 N1 P1	1.67884	0.010297907	-0.870328703
5	LPC(18 : 0) + H	LPC(18 : 0) + H	LPC	(18 : 0)	C26 H55 O7 N1 P1	1.57303	0.034060664	-1.147680261
6	LPC(20 : 4) + H	LPC(20 : 4) + H	LPC	(20 : 4)	C28 H51 O7 N1 P1	1.61395	0.023265476	-0.549852755
7	PE(16 : 0p/18 : 1) + H	PE(34 : 1p) + H	PE	(16 : 0p/18 : 1)	C39 H77 O7 N1 P1	1.64662	0.016093919	1.531453663
8	PE(38 : 6) + H	PE(38 : 6) + H	PE	(38 : 6)	C43 H75 O8 N1 P1	1.65057	0.015315495	-0.898920166
9	TG(15 : 0/16 : 0/16 : 0) + Na	TG(47 : 0) + Na	TG	(15 : 0/16 : 0/16 : 0)	C50 H96 O6 Na1	1.59647	0.027631258	0.747616493
10	TG(16 : 0/12 : 0/13 : 0) + NH_4_	TG(41 : 0) + NH_4_	TG	(16 : 0/12 : 0/13 : 0)	C44 H88 O6 N1	1.56416	0.036667833	1.376598918

**Table 5:** Detailed differences in lipid metabolites between the ATP9A-OV and plv-NC groups in negative mode.

No.	Lipid ion	Lipid group	Class	Fatty acid	Ion formula	VIP	*T*-test	Log_FC(ATP9A-OV/plv-NC)
1	LPC(16 : 0) + HCOO	LPC(16 : 0) + HCOO	LPC	(16 : 0)	C25 H51 O9 N1 P1	1.42619	0.02839175	-0.956682794
2	PC(16 : 0/18 : 2) + HCOO	PC(34 : 2) + HCOO	PC	(16 : 0/18 : 2)	C43 H81 O10 N1 P1	1.40826	0.033403463	-0.777315557
3	PC(16 : 0/20 : 4) + HCOO	PC(36 : 4) + HCOO	PC	(16 : 0/20 : 4)	C45 H81 O10 N1 P1	1.45251	0.020556433	-0.64073783
4	PC(18 : 0/18 : 2) + HCOO	PC(36 : 2) + HCOO	PC	(18 : 0/18 : 2)	C45 H85 O10 N1 P1	1.4845	0.011801161	-0.732314818
5	PC(16 : 0/22 : 6) + HCOO	PC(38 : 6) + HCOO	PC	(16 : 0/22 : 6)	C47 H81 O10 N1 P1	1.38346	0.030681364	-0.753866237
6	MGDG(47 : 12) + HCOO	MGDG(47 : 12) + HCOO	MGDG	(47 : 12)	C57 H85 O12	1.52892	0.004986124	-0.722153703

## Data Availability

The datasets generated and analyzed during the current study are available from the corresponding authors on reasonable request.
